# Characterization of the *tilt (tt)* phenotype in *Drosophila melanogaster*

**DOI:** 10.17912/micropub.biology.000788

**Published:** 2023-04-29

**Authors:** Arno Houtman, Samuel Gruber, Hailey Reisert, Mina Amini, Caroline Fiore, Paula Gonzalez, Veronica Han, Aeva Jazic, Mie Kusupholnand, Max Miller, Jiung Nam, Ziqin Wang, Yang Yu, Peter Dong, Allen S. W. Oak, Arun Sharma, Eric P Spana

**Affiliations:** 1 Duke Kunshan University, Kunshan, Jiangsu, China; 2 Department of Biology, Duke University, Durham, North Carolina, United States; 3 Department of Dermatology, Hospital of the University of Pennsylvania, Philadelphia, Pennsylvania, United States; 4 Department of Biomedical Sciences; Board of Governors Regenerative Medicine Institute; and Smidt Heart Institute, Cedars-Sinai Medical Center, Los Angeles, California, United States

## Abstract

In the early 20th century, Calvin Bridges and Thomas Morgan identified a number of spontaneous mutations that displayed visible phenotypes in adult flies and subsequent analysis of these mutations over the past century have provided fundamental insights into subdisciplines of biology such as genetics, developmental, and cell biology. One of the mutations they identified in 1915 was named *tilt* ( *tt* ) and was described by Bridges and Morgan as having two visible phenotype characteristics in the wing. The wings were “held out at a wider angle from the body” and had a break in wing vein L3. Subsequent analysis of the *tilt* phenotype identified another phenotype: the wings were missing a varying number of campaniform sensilla on L3. Though Bridges and Morgan provided an ink drawing of the wing posture phenotype, only the vein and campaniform sensilla loss images have been published. Here we confirm and document the *tilt* phenotypes that have been previously described. We also show the penetrance of these phenotypes: the vein break and the distinct outward wing posture have decreased since its discovery.

**Figure 1. Characterization of the tilt phenotype:**
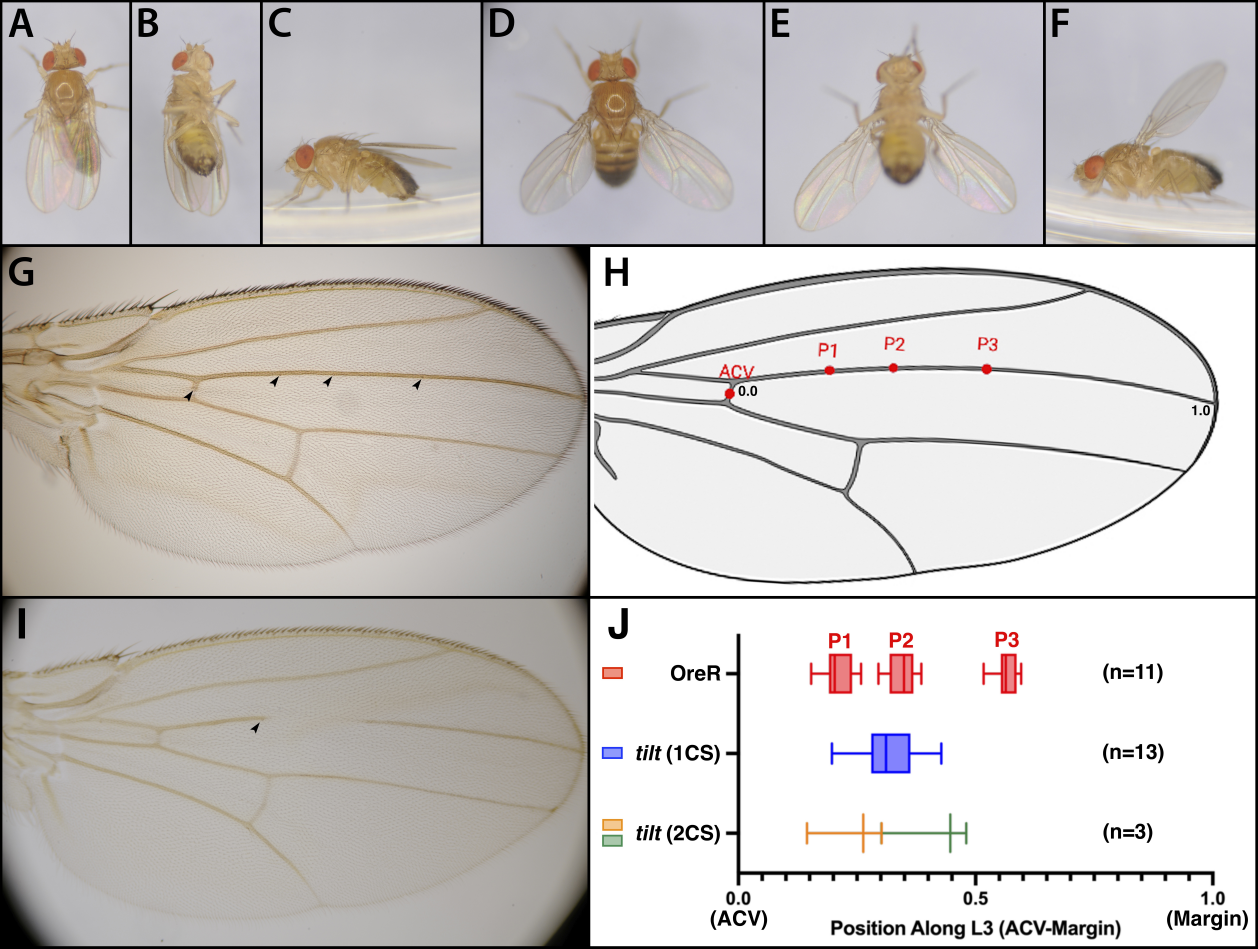
The wing position phenotype of live OreR and homozygous *tilt* male flies from dorsal **(A, D)** , ventral **(B, E)** , and lateral view **(C, F)** is shown. OreR Wings are held tight to the body, while the *tilt* wing is held out at 45° from the body in both the dorsal/ventral and anterior/posterior directions. The vein phenotype of male OreR wing **(G)** and male *tilt* wing **(I)** shows the region of the L3 vein lost in *tilt* . The location of the campaniform sensilla is shown by arrowheads. The *tilt* wing is missing two out of three L3 sensilla. **(H)** Average observed campaniform sensilla (L3-1, L3-2, L3-3) positions (P1-3) from n=11 OreR adult male Drosophila wings visualized on a wing diagram. The position of ACV sensilla was placed in the center of the anterior cross vein. **(J)** L3 sensilla position was represented as a ratio between the distance of each sensillum from the ACV over the total distance of the wing, measured from the ACV (0.0) to the wing margin (1.0). The observed positions (P1, P2, P3) for L3-1, L3-2, L3-3 sensilla are shown for 11 OreR male wings (red). The sensilla positions of 13 *tilt* males that had a single sensilla (blue) and 3 *tilt* males that had two sensilla (proximal sensilla in gold, distal sensilla in green) are shown below. The scale of panels H and J are approximately matched.

## Description

At the beginning of the twentieth century Calvin Bridges and Thomas Hunt Morgan published analyses of 62 mutant characteristics located on the third chromosome (Bridges and Morgan, 1923) . Of these 62 mutant characteristics, only 21 are mapped to a transcription unit and one of the remaining unmapped mutants is *tilt* ( *tt* ) *.* Bridges and Morgan originally described the *tilt* mutation as defined by wings that were held out and tilted upward and that this phenotype was found in about 75% of the *tilt* flies. They also found approximately 90% of *tilt* wings had breaks in the L3 vein. Waddington described that the L3 vein would originally emerge complete, but the central section would disappear during the pupal contraction period (Waddington, 1940) . Decades later, Thompson *et al.* found the *tilt* mutation also affected the formation of campaniform sensilla along the L3 vein, with mutants never forming all four of the L3 sensilla: ACV, L3-1, L3-2, or L3-3 (Thompson Jr. *et al.* , 1982). *tilt* was placed in the group of mutations that lacked all or some of longitudinal veins (Diaz-Benjumea and Garcia-Bellido, 1990) . Within this group however, *tilt* was found to only have additive effects with the other mutations. Later *tilt* was found to have no effect on phenotypes generated by ectopic rhomboid expression, though rhomboid expression was lost in disks and pupae in the L3 region in *tilt* mutants (Sturtevant and Bier, 1995) . Since the discovery of *tilt ^1^* by Calvin Bridges in August 1915, no second allele of tilt has been identified.

In a precursor to mapping, we reanalyzed the phenotype of tilt ^1^ homozygotes (hereafter referred to as ‘ *tilt* mutants’ or simply ‘ *tilt* ’) to identify which phenotypes could be accurately scored in a complementation test (ie, wing position, vein loss, and/or sensilla loss). Similar to the description provided by Bridges and Morgan we found that the *tilt* flies not only held their wings out from the body, but also dorsally in an elevated “up and out” manner compared to wild-type flies (Fig 1. A-F). However, the percentage of *tilt* flies that showed these characteristics had decreased from 75% to 50%. The *tilt* phenotype differs from other ‘held out’ phenotypes such as the *os-1* allele of *unpaired-1* ( *upd1 ^os-o^* ) (Johnstone *et al.* , 2013); *aeroplane* (now identified as *teashirt* , Quelprud, 1931; Soanes and Bell, 2001); or *taxi* (Collins, 1928; Egoz-Matia *et al.* , 2011) in that only *tilt* holds the wings up as seen in Fig 1 F. The other mutations present similar phenotypes to Fig 1 E, but the wings are kept horizontal to the walking surface.

An even more striking phenotypic change was the L3 vein break; only about 1% of *tilt* wings showed a break compared to 90% of wings when the mutant was first isolated (Fig.1 I). For *tilt* wings, the most penetrant phenotypic difference was campaniform sensilla loss along the third longitudinal wing vein (L3) and anterior cross vein (ACV). We scored 61 wings from *tilt* males and found that only 6.56% had the ACV sensilla, 26.23% had P1, 42.62% had P2, and 4.92% had P3. In contrast, the 56 wings scored from *OreR* males had all four sensilla (Fig.1 G, I, H). For comparison, Thomson *et al.* did not count ACV sensilla specifically, but did record their observations for the three sensilla distal to it: ~83% of their flies had no distal sensilla, and ~16% had one, and ~1% had two sensilla. They did not record finding any wings with 3 distal sensilla. As with the previous phenotypes, the number of sensilla we scored was not quite as severe: 36.8% of *tilt* wings had zero distal sensilla (25/68 wings), 58.8% had a single sensilla (40/68), and 4.4% had two sensilla (3/68). We did not observe a single wing with three distal sensilla. Another observation was that sensilla, when present in the *tilt* wings, were often mispositioned. We measured the position of each sensilla along the L3 vein in both OreR and *tilt* wings. Specifically, we found three regions/positions (P1, P2, and P3) for the L3-1, L3-2, and L3-3 sensilla in *OreR* males, whereas the *tilt* sensilla were more scattered and commonly lie in the position between the P1 and P2 positions of OreR ( Fig. 1 H, J ). The mispositioning of sensilla might lead to misidentification of sensilla L3-1 and L3-2 in *tilt* wings where only a single sensilla is present but might reside in a novel position. Examination of the projections from the neurons of these sensilla might lead to an unambiguous determination (Palka, 1993) .

Our analysis confirms the *tilt* phenotype as described by Bridges and Morgan as well as Thompson *et al.* However, there are two major differences in comparing the analysis. One is the break in the L3 vein which was previously present in the vast majority of *tilt* wings but is now barely seen. The second is the outstretched wing phenotype which also occurs slightly less frequently than previously recorded. Each of these changes could result from the *tilt* stock accumulating modifiers over the last 40 to 100 years. Our finding is that not only are sensilla lost in *tilt* flies but when present, they are located elsewhere than the normally defined positions seen in the *OreR* wild-type stock. Instead, they are proximally distributed all along the wing vein. The position shift and loss of sensilla may be due to an underlying patterning defect in L3. Overall this recharacterization of *tilt* serves to redefine the current *tilt* phenotype and shows that of the various phenotypes *tilt* mutants possess, the loss of campaniform sensilla is the most robust and useful for complementation mapping.

## Methods

***D. melanogaster* stocks and genetics:** *D. melanogaster* was cultured on cornmeal molasses agar food (Archon Scientific) using standard techniques. All experiments were performed on adult males. Adult females show similar penetrance for wing posture and vein breaks. Wild-type OreR flies were a lab stock while *tilt* flies ( *tt ^1^ wo ^1^* ) were ordered from the Bloomington Drosophila Stock Center at Indiana University.

**Adult wing imaging and live photography:** Wings were removed and placed on glass slides (Globe Scientific). The wings were then washed in 100% ethanol, air dried, and mounted in ~20µL of Euparal (BioQuip Products, Inc.). The Euparal hardened under 22 mm square coverslips (VWR) on a slide warmer for ~24 hours before imaging with a Nikon D300S camera coupled to a Leica DMRB microscope. Adult male wings fit nicely in the field of view, while female wings are a little too big to fit both the wing tip and alula in frame. To image wing posture, live OreR or *tilt* males were placed in a 35 mm culture dish and photographed through the lid at 80X magnification using a Leica MZ16F stereomicroscope coupled to a Nikon D300S camera.

**Sensilla scoring:** Images of mounted wings of both OreR and *tilt* flies were examined in ImageJ. Sensilla position was measured as a ratio between the distance of each sensillum from the ACV over the total distance of the wing, measured from the ACV to the wing margin. Data was visualized using Prism by Graphpad Software.

## Reagents

**Table:** 

Reagent type	Designation	Identifier	References or source	Additional information
Genetic reagent ( *D. melanogaster* )	*tilt*	BDSC 623	Bloomington Drosophila Stock Center	* tt ^1^ wo ^1^*
Genetic reagent ( *D. melanogaster* )	OreR	N/A	N/A	Laboratory strain
Software	BioRender	N/A	https://biorender.com/	
Software	ImageJ	N/A	https://imagej.nih.gov/ij/download.html	ImageJ bundled with Java 1.8.0_172
Software	Prism	N/A	https://www.graphpad.com/features	
Chemical	Euparal mounting media	Cat# 6372A	BioQuip Products Inc.	
Fly food	Molasses	N/A	Archon Scientific	Molasses Food in Narrow PS Vials, 10 mL, Lot B222020
